# High-Precision Vital Signs Monitoring Method Using a FMCW Millimeter-Wave Sensor

**DOI:** 10.3390/s22197543

**Published:** 2022-10-05

**Authors:** Mingxu Xiang, Wu Ren, Weiming Li, Zhenghui Xue, Xinyue Jiang

**Affiliations:** 1 School of Integrated Circuits and Electronics, Beijing Institute of Technology, Beijing 100081, China; 2 School of Information and Electronics, Beijing Institute of Technology, Beijing 100081, China

**Keywords:** human vital signs detecting, FMCW radar sensor, impulse denoising, spectral estimation decision

## Abstract

The method of using millimeter-wave radar sensors to detect human vital signs, namely respiration and heart rate, has received widespread attention in non-contact monitoring. These sensors are compact, lightweight, and able to sense and detect various scenarios. However, it still faces serious problems of noisy interference in hardware, which leads to a low signal-to-noise ratio (SNR). We used a frequency-modulated continuous wave (FMCW) radar sensor operating at 77 GHz in an office environment to extract the respiration and heart rate of a person accustomed to sitting in a chair. Indeed, the proposed signal processing includes novel impulse denoising operations and the spectral estimation decision method, which are unique in terms of noise reduction and accuracy improvement. In addition, the proposed method provides high-quality, repeatable respiration and heart rates with relative errors of 1.33% and 1.96% on average compared with the reference values measured by a reliable smart bracelet.

## 1. Introduction

Using the millimeter wave Doppler sensor [1] to non-contact monitor the vital signs of human targets [1,2,3] can be applied to many application scenarios where contact methods are not applicable. These scenarios include special patient monitoring in medical care [4], such as burn patients [5], newborns [6], infectious disease patients, and early warning of emergencies such as home healthcare for the elderly [7] and the avoidance of driver fatigue [8], etc. Doppler radar sensors can detect the movement of human targets by measuring the low Doppler variation that affects the received backscattered signals [1]. They are focused on two different systems: ultra-wide band (UWB) radars and continuous wave (CW) radars. Compared with the UWB radar, the FMCW radar relies on phase information to obtain target displacement information with higher detection accuracy. In addition, its non-broadband characteristic achieves low power consumption.

At present, increasing the SNR level of the signal to improve the accuracy of the respiration and heart rate is a research focus of numerous researchers. Multiple channels of the MIMO radar were adopted to collect data, as covered in paper [9], and the SNR has been improved by channel diversity. This puts forward requirements for the hardware system. At the same time, the maximum ratio combination algorithm based on adaptive continuous wavelet transform was proposed to improve the precision of heart rate detection. An adaptive noise cancellation technique combined with the polynomial fitting was proposed by the authors of [10], to offset the influence of random human movement on the extraction of small vital-sign signals. Meanwhile, a new discrete cosine transform was introduced to improve detection accuracy. H.-I. Choi et al. defined the time phase coherence index as a new distance bin selection method [11]. By analyzing the minute phase shift of each range bin, they selected the range box containing the accurate heartbeat signal to extract it and then improved the estimate accuracy. A method of reconstructing heartbeat signals by their harmonics and using the Kalman filter to improve the quality of the heartbeat waveform further was proposed by the authors of [12], which avoided the influence of respiratory harmonics on heartbeat signals to a certain extent. M. Arsalan et al. introduced the Kalman filter to track the heartbeat signal. They used an adaptive band-pass filter to extract the heart rate [13], which continuously reduced the bandwidth of the band-pass filter and approximated its final center frequency as the actual heart rate to improve its accuracy.

This paper demonstrates the feasibility of measuring human vital-sign signals using a 77 GHz FMCW sensor. We extracted the respiratory and heart rate using the phase information of the IF signal, a mixture of transmitted and received waves. Then, an algorithm called Iterative VMD Wavelet-Interval-Thresholding was proposed to reduce the noise in the measurement process. The experimental results show that the proposed algorithm can improve the SNR of respiration and heartbeat signals by 1.89 dB and 1.44 dB. Section 2 of this paper describes the theoretical model of vital signs signals from the FMCW radar’s perspective. It shows the flowchart of the whole signal process, focusing on the principle and steps of the noise reduction algorithm and the rate estimation algorithm. The experimental setups are presented in Section 3, and the verification by experimental results is drawn in Section 4. Finally, Section 5 provides the discussion and the conclusion is provided in Section 6.

## 2. Principles

### 2.1. Signal Model of FMCW Radar

We analyzed the principle of the FMCW radar detecting human vital-sign signals in our previous paper [14]. The modulated mode of the transmitted wave is a sawtooth wave with the slope as K and the chirp duration as $T_{c}$. The expression of the mixing signal output by channel I and channel Q at the receiving end of the radar can be expressed in theory as follows:

$$I\left(t\right)=A_{r}\cos\left(\frac{4\pi x\left(t\right)}{\lambda_{\max}}+\frac{4{\pi R}_{0}}{\lambda_{\max}}+2\pi K\tau t+\Delta \phi \right),$$

(1) $$Q\left(t\right)=A_{r}\sin\left(\frac{4\pi x\left(t\right)}{\lambda_{\max}}+\frac{4{\pi R}_{0}}{\lambda_{\max}}+2\pi K\tau t+\Delta \phi^{\prime }\right),$$

where $A_{r}$ is the amplitude of the beat signal; $\tau \approx 2R_{0}/c$ is the time delay caused by the distance between the measured target and the radar marked as $R_{0}$; $\lambda_{\max}=c/f_{c}$ is the wavelength corresponding to the chirp starting frequency $f_{c}$; Δϕ and Δϕ′ are the phase noise of channel I and Q, respectively; $x\left(t\right)$ is the slight time-varying displacement caused by cardiopulmonary activities containing vital signs such as breathing and heartbeat or other movements. The subsequent work is to sample and process the above signals.

### 2.2. The Proposed Signal Processing Algorithm Chain

**Figure 1:**
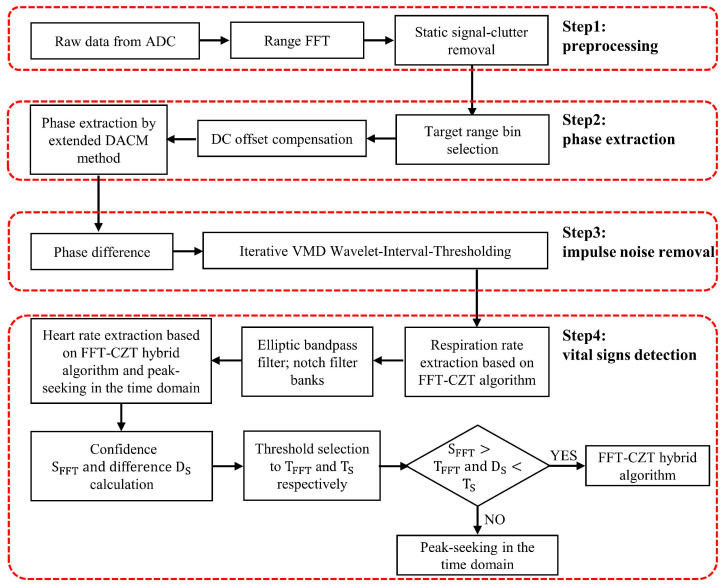
The proposed signal processing algorithm chain.

Figure 1 shows the proposed signal-processing flowchart, where the processing signal is the IF signal output by the I and Q channels of the FMCW millimeter wave sensor. The process comprises four steps: signal preprocessing, phase extraction in range window, impulse noise removal, and vital signs detection. 

After the IF signal is sampled by ADC, the data acquisition board exports the sampled data to form a two-dimensional matrix. The sampled data in each chirp constitute the matrix’s columns, and the matrix’s rows are composed of different frames. It should be noted that we set only one chirp in each frame. The sampled data are preprocessed mainly through static signal-clutter removal to eliminate the background interference in the environment for the position information of the measured target. The fast Fourier transform (FFT) is applied to the column vectors of the matrix successively to obtain the precise range window of the measured target, and then we further extract the phase of each frame in the range window. The vital signs of the target can be obtained from the phase information. To eliminate the influence of the DC components of the I and Q channels on phase extraction, the DC offset compensation operation is carried out to obtain the original phase. The phase difference operation plays a significant role in enhancing the heartbeat signal components. Then the iterative VMD Wavelet-Interval-Thresholding algorithm is proposed to filter the impulse noise generated by the differential process. In the vital signs detection stage, respiration and heart rates are extracted successively. Among them, the respiration rate is obtained by the FFT-CZT mixed spectrum estimation algorithm, while the heart rate is estimated by FFT-CZT and peak-seeking in the time domain method separately. Then we combine the confidence degree for thresholding judgment, and one of the results obtained by the two methods above is selected as the final result. The details of each step are elaborated in the sections as follows.

#### 2.2.1. Range FFT and Static Signal-Clutter Removal

**Figure 2:**
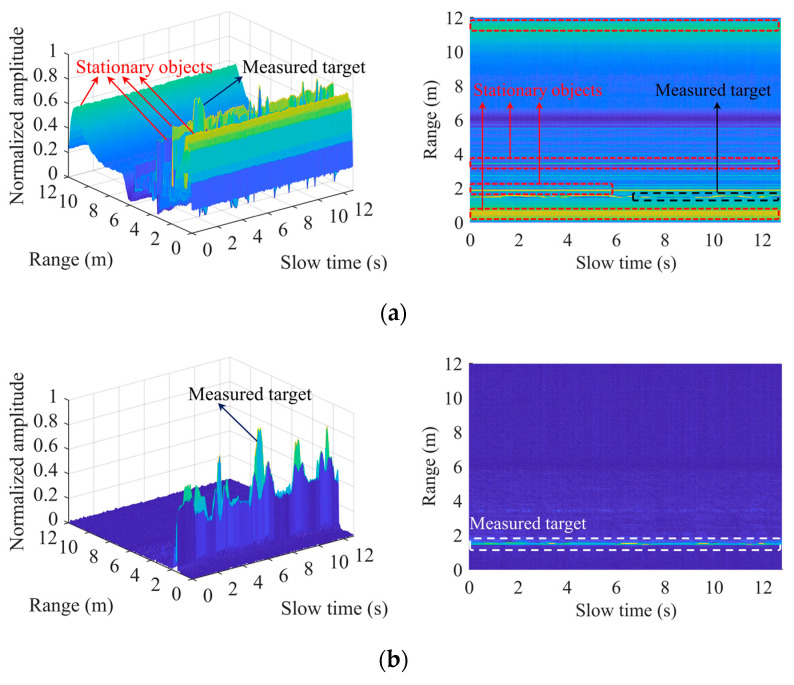
Range-FFT: (**a**) before static signal-clutter removal; (**b**) after static signal-clutter removal.

The 1D-FFT in the distance dimension is performed on the two-dimensional matrix formed by sampling the IF signal to obtain the range slow-time matrix. Due to the complex background in the actual detection environment, the reflected static clutter signals will affect the selection of the target range bin and introduce some phase noise, which is not conducive to the extraction of the cardiopulmonary information. Therefore, the interference of static clutter should be removed. In the same test scenario, the clutter signals caused by other stationary objects basically remain unchanged with slow time, so subtracting the mean value of the slow-time dimension of each range window from the data in the slow-time dimension corresponding to each range bin can remove the background noise and retain the movement information of the measured target. Assuming that $y_{0}$ is the two-dimensional matrix composed of the original collected data, the matrix after the removal of static clutter signals can be calculated as follows:

(2) $$y\left[m,n\right]=y_{0}\left[m,n\right]-\frac{1}{N_{\mathrm{frames}}}\overset{N_{\mathrm{frames}}}{\underset{n=1}{\sum }}y_{0}\left[m,n\right],$$

where m = 1, 2, 3, …, $N_{\mathrm{samples}}$; n = 1, 2, 3, …, $N_{\mathrm{frames}}$. $N_{\mathrm{samples}}$ means the number of sampling points of each chirp; and $N_{\mathrm{frames}}$ means the number of frames. Figure 2 is the schematic diagram of the distance slow-time matrix before and after the static signal-clutter removal operation. Figure 2b shows the range bin where the measured target is located, which will be further processed later to extract the phase.

#### 2.2.2. DC Offset Compensation

**Figure 3:**
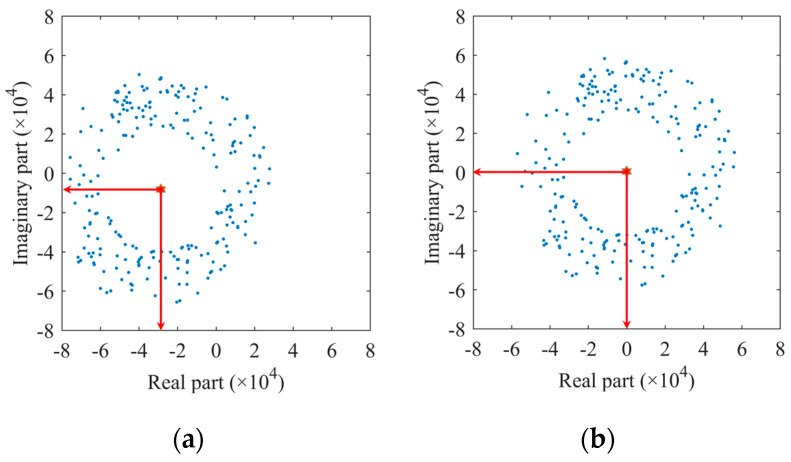
The complex data at the selected range bin: (**a**) before DC offset compensation; (**b**) after DC offset compensation.

Because of the limitations of the manufacturing process of the hardware circuit in the receiver, the sensor used may have problems of insufficient mixer isolation or incomplete matching between the receiving antenna and the transmitting antenna. Therefore, a certain DC offset still exists after solving the static signal clutter caused by the fixed objects in the detection environment. DC offset will introduce more phase noise, resulting in the phase’s distortion extraction of the echo signal by the arctangent operation. The extracted phase can be expressed as:

(3) $$\Psi =\arctan\left[\frac{Q\left(t\right)+{\mathrm{DC}}_{Q}}{I\left(t\right)+{\mathrm{DC}}_{I}}\right]\neq \arctan\left[\frac{Q\left(t\right)}{I\left(t\right)}\right],$$

where ${\mathrm{DC}}_{I}$, ${\mathrm{DC}}_{Q}$ are the real and imaginary parts of the DC offset, respectively. In the complex plane, because of the existence of the DC offset component, the constellation center of the complex signal in the selected target range bin moves from the origin to (${\mathrm{DC}}_{I}$, ${\mathrm{DC}}_{Q}$), as shown in Figure 3a. In order to shift the constellation to the origin, we use the non-linear least square estimation (NLLS) proposed by the authors of [15] to estimate the center and radius of the constellation. The DC offset compensation can be realized by successively subtracting the estimated center point position from each sampling point corresponding to the slow-time dimension in the selected range bin. Figure 3b shows the results after compensation.

#### 2.2.3. Phase Extraction and Difference Operation

After DC offset compensation, we need to extract the phase information of the complex data. Using the arctangent algorithm to extract the phase will have a breakpoint problem, leading to phase drift exceeding ±π. Assuming that the real and imaginary parts of the complex data above are expressed as I(n) and Q(n), respectively, we apply the extended differentiate and cross multiply (DACM) algorithm [16] to extract the phase, to avoid the phase drifts mentioned above, as follows:

(4) $$\Psi \left(n\right)=\overset{n}{\underset{i=2}{\sum }}\frac{I\left(i\right)\cdot \left[Q\left(i\right)-Q\left(i-1\right)\right]-Q\left(i\right)\cdot \left[I\left(i\right)-I\left(i-1\right)\right]}{I{\left(i\right)}^{2}+Q{\left(i\right)}^{2}}.$$

**Figure 4:**
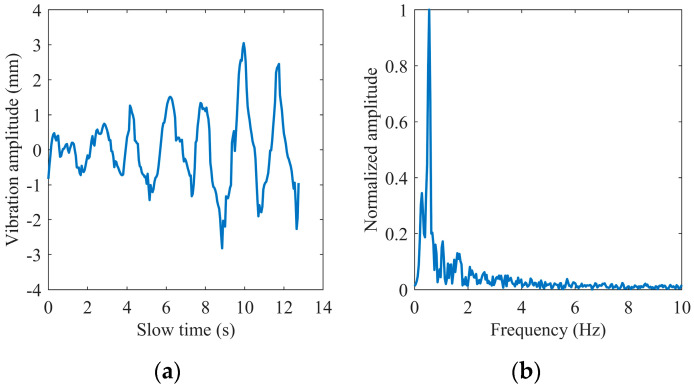
Extracted phase by the extended DACM: (**a**) in the time domain; (**b**) in the frequency domain.

**Figure 5:**
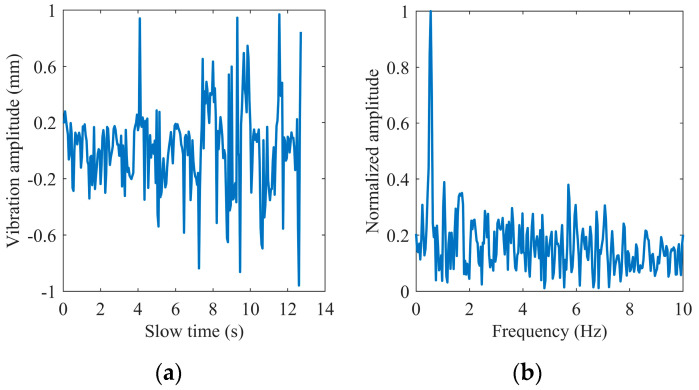
Phase difference: (**a**) in the time domain; (**b**) in the frequency domain.

Figure 4 shows an example of extracting the phase of a complex signal using the extended DACM algorithm. We transform the extracted phase into the waveform of chest vibration amplitude varying with slow time and obtain its spectrum information through FFT. We found that the heartbeat frequency is covered in the spectrum in Figure 4b because the chest fluctuation caused by the heartbeat is very weak relative to breathing. The first-order phase difference operation is adopted to enhance the heartbeat signal. Figure 5 shows the time domain waveform and frequency domain spectrum of the differential signal, where the spectral amplitude in the heart rate range is increased. The generated impulse components are removed in the following steps.

#### 2.2.4. Iterative VMD Wavelet-Interval-Thresholding

Iterative VMD Wavelet-Interval-Thresholding is mainly used to remove the impulse noise generated after the phase difference operation. This process refers to the EMD-CIIT described in paper [17]. Compared with the empirical mode decomposition (EMD) algorithm, the variational mode decomposition (VMD) algorithm also decomposes the original signal into a series of signal components, which is called the intrinsic mode function (IMF). However, the latter is supported by sufficient mathematical theory [18]. Furthermore, the defect of modal aliasing is avoided by decomposing the signal into a certain number of discrete signal components, in which each IMF has different center frequencies and limited bandwidth. Assuming that VMD decomposes the signal into K IMFs, the kth IMF is recorded as $u_{k}\left(t\right)$, and its center frequency is $\omega_{k}$; the goal of the algorithm is to minimize the sum of the estimated bandwidth of each mode, that is:

(5) $$\underset{\left\{u_{k}\right\},\left\{\omega_{k}\right\}}{\min}\left\{\overset{K}{\underset{k=1}{\sum }}∥-{j\omega }_{k}{\left[\left(\delta \left(t\right)+\frac{j}{\pi t}\right)*u_{k}\left(t\right)\right]}^{\prime }e^{-{j\omega }_{k}t}{∥}_{2}^{2}\right\},$$

where $\left\{u_{k}\right\}$ means $\left\{u_{1},\ldots ,u_{K}\right\}$ and $\left\{\omega_{k}\right\}$ means $\left\{\omega_{1},\ldots ,\omega_{K}\right\}$. In order to solve this constrained variational problem, the quadratic penalty term α and Lagrangian multiplier $\lambda \left(t\right)$ are introduced, which can transform the problem into an unconstrained variational problem as follows:

(6) $$L\left(\left\{u_{k}\right\},\left\{\omega_{k}\right\},\lambda \left(t\right)\right)=\alpha \overset{K}{\underset{k=1}{\sum }}∥-{j\omega }_{k}{\left[\left(\delta \left(t\right)+\frac{j}{\pi t}\right)*u_{k}\left(t\right)\right]}^{\prime }e^{-{j\omega }_{k}t}{∥}_{2}^{2}\;+∥f\left(t\right)-\overset{K}{\underset{k=1}{\sum }}u_{k}\left(t\right){∥}_{2}^{2}+\langle \lambda \left(t\right),f\left(t\right)-\overset{K}{\underset{k=1}{\sum }}u_{k}\left(t\right)\rangle .$$

Then, $\left\{\omega_{k}\right\}$ and $\left\{u_{k}\right\}$ are obtained by the alternate direction method of multipliers (ADMM), which can be described in the frequency domain:

$$U_{k}^{n+1}\left(\omega \right)=\frac{F\left(\omega \right)-\sum_{i\neq k}U_{i}\left(\omega \right)+\frac{\;\hat{\lambda }\left(\omega \right)}{2}}{1+2\alpha {\left(\omega -\omega_{k}\right)}^{2}},\;\omega_{k}^{n+1}=\frac{\int_{0}^{\infty }\omega {\left|U_{k}\left(\omega \right)\right|}^{2}d\omega }{\int_{0}^{\infty }{\left|U_{k}\left(\omega \right)\right|}^{2}d\omega },$$

(7) $$\hat{\lambda }{}^{n+1}\left(\omega \right)=\;\hat{\lambda }{}^{n}\left(\omega \right)+\tau \left(F\left(\omega \right)-\overset{K}{\underset{k=1}{\sum }}U_{k}^{n+1}\left(\omega \right)\right).$$

The algorithm can be divided into the following steps: first, initialize $\left\{U_{k}^{1}\right\}$, $\left\{\omega_{k}^{1}\right\}$, $\lambda^{1}$, and n, which is the number of loops; then update $\left\{U_{k}\right\}$, $\left\{\omega_{k}\right\}$, and λ according to (7); repeat the previous step until the following constraints are satisfied:

(8) $$\overset{K}{\underset{k=1}{\sum }}\frac{{\left|\left|U_{k}^{n+1}-U_{k}^{n}\right|\right|}_{2}^{2}}{{\left|\left|U_{k}^{n}\right|\right|}_{2}^{2}}<\varepsilon .$$

We set α as 2000, and τ = 0, which means that the signal is restored without introducing additional noise; at the same time, we set ε to $10^{-7}$, which refers to the convergence tolerance. 

**Figure 6:**
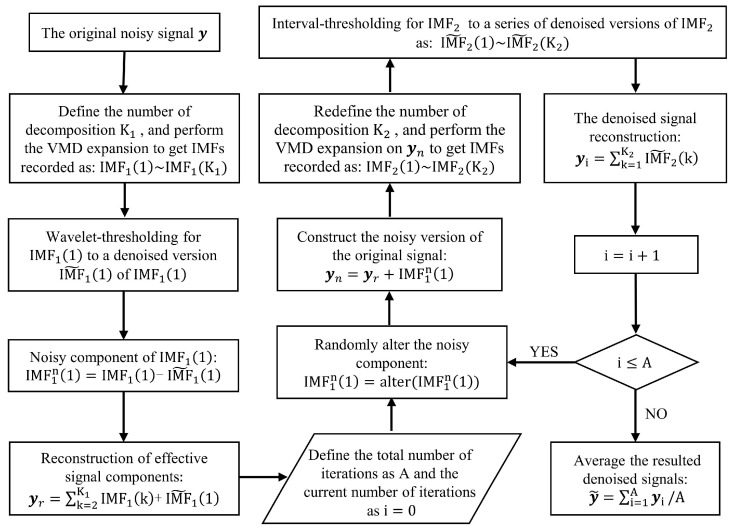
The algorithm chain of Iterative VMD Wavelet-Interval-Thresholding.

According to the above theoretical inference, VMD decomposes the original signal into the sum of multiple IMFs, where each IMF is a narrow-band amplitude and frequency modulation (AM-FM) signal. After comparing the energy and spectrum of each IMF with the original signal, we find that the first IMF basically does not contain useful signal components, so it is considered to contain noise components, and the remaining IMFs are regarded as signal components. The noise reduction process is shown in Figure 6, and the specific steps are as follows:

*(1) VMD expansion:* Perform the VMD decomposition of the original noisy signal y to obtain ${\mathrm{IMF}}_{1}\left(1\right)\sim {\mathrm{IMF}}_{1}\left(K_{1}\right)$, in which the number of decomposition times is $K_{1}$. The selection of $K_{1}$ refers to the optimal number of decomposition modes in the improved VMD algorithm [19], in which the cross-correlation number is used to represent the correlation degree between the noise component and the signal component. In order to facilitate subsequent operations, the first IMF is taken as the noise component and expressed as ${\mathrm{IMF}}_{1}\left(1\right)$, while the remaining components are reconstructed to obtain the signal component and expressed as ${\mathrm{IMF}}_{1}\left(h\right)$. Then, the cross-correlation coefficient of the two can be expressed as:

(9) $$\rho_{c}=\frac{R_{1,h}\left(1\right)}{\sqrt{R_{1,1}\left(0\right)R_{h,h}\left(0\right)}}=\frac{\sum_{n=1}^{N}{\mathrm{IMF}}_{1}\left(n,1\right){\mathrm{IMF}}_{1}\left(n-1,h\right)}{\sqrt{R_{1,1}\left(0\right)R_{h,h}\left(0\right)}},$$

where $R_{1,1}\left(0\right)$ and $R_{h,h}\left(0\right)$ are the autocorrelation functions of noise component ${\mathrm{IMF}}_{1}\left(1\right)$ and signal component ${\mathrm{IMF}}_{1}\left(h\right)$, respectively. $R_{1,h}\left(1\right)$ is the cross-correlation function of the two components mentioned above, in which the time difference is set as 1 and then the correlation is maximum. Next, calculate the cross-correlation coefficient for each decomposition number, ranging from 2 to 8. This range is because when the number is too large, a certain modal component may exist in multiple modal components, and then the spectrum fracture phenomenon will occur. Then, select the decomposition number corresponding to the smallest cross-correlation coefficient as $K_{1}$.

(*2) Wavelet-thresholding:* The first IMF obtained, which is called ${\mathrm{IMF}}_{1}\left(1\right)$, is denoised by wavelet-thresholding. The noise reduction process is summarized in the following steps:

- Select the appropriate wavelet basis function and determine the layers of the wavelet decomposition;
- Obtain a set of wavelet decomposition coefficients in the wavelet domain by wavelet decomposition of ${\mathrm{IMF}}_{1}\left(1\right);$

- Set the threshold, and use the soft threshold function to process the wavelet coefficients.

The wavelet coefficients larger than the threshold are retained as the components caused by the target signal. In contrast, the wavelet coefficients smaller than the threshold are discarded as the components caused by the noise signal. Assuming that the threshold is called T and the coefficient is marked as x, it can be described as:

(10) $$f\left(x\right)=\left\{\begin{array}{ll} \mathrm{sgn}\left(x\right)\left(\left|x\right|-T\right), & \left|x\right|>T\\ 0, & \left|x\right|\leq T \end{array}\right..$$

- Reconstruct the signal by the processed wavelet coefficients to obtain the denoised signal ${\tilde{\mathrm{IMF}}}_{1}\left(1\right)$.

We choose the Symlets wavelet function as the basis function because it meets the compactness and regularity with good symmetry, reducing the phase distortion caused by signal analysis and reconstruction to a certain extent. This algorithm selects the sym4 wavelet, whose support length is 7 and the number of vanishing moments is 4. At the same time, we set the layers of wavelet decomposition to 4, and the threshold is determined by using the ddencmp function in MATLAB. The filtered part is taken as the redefined noise component ${\mathrm{IMF}}_{1}^{n}\left(1\right)$, and ${\tilde{\mathrm{IMF}}}_{1}\left(1\right)$ is added to the remaining IMFs to form the redefined signal component, which is marked as $y_{r}$;

*(3) Randomly alter:* Considering the randomness of the noise contained, we chose to randomly alter the noise component ${\mathrm{IMF}}_{1}^{n}\left(1\right)$ to obtain a different version of the noise component. It means that the sequence ${\mathrm{IMF}}_{1}^{n}\left(1\right)$ is circularly shifted randomly, that is, the number of cycle-shift-right is randomly selected;

*(4) Signal reconstruction:* Add the noise component ${\mathrm{IMF}}_{1}^{n}\left(1\right)$ obtained by step (3) and the redefined signal component $y_{r}$ obtained by step (2) to obtain the equivalent version of the original noisy signal, which is marked as $y_{n}$;

*(5) Interval-thresholding:* Perform the VMD decomposition of $y_{n}$ to obtain ${\mathrm{IMF}}_{2}\left(1\right)\sim {\mathrm{IMF}}_{2}\left(K_{2}\right)$, in which the number of decomposition times is $K_{2}$. Then perform Interval-thresholding on them successively to obtain ${\tilde{\mathrm{IMF}}}_{2}\left(1\right)\sim {\tilde{\mathrm{IMF}}}_{2}\left(K_{2}\right)$, and add all of them to obtain the noise reduction version $y_{i}$. The following is the noise reduction basis of Interval-thresholding.

**Figure 7:**
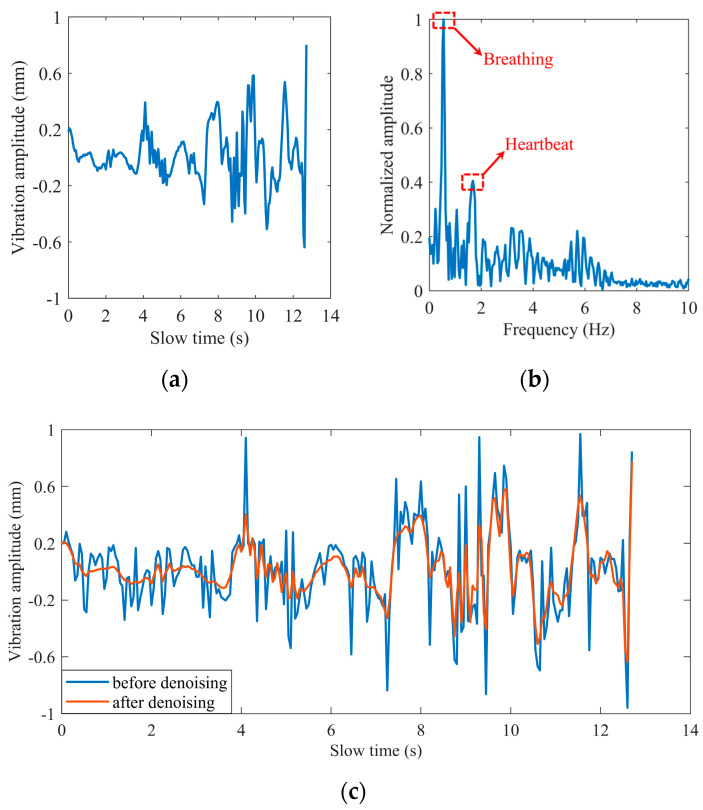
The impulse noise removal: (**a**) in the time domain; (**b**) in the frequency domain; (**c**) waveform comparison before and after denoising.

Considering the general characteristics of the IMF obtained by VMD decomposition, we define the waveform between adjacent zero crossings as a unit of the IMF. These characteristics include that the difference between the number of extreme points and zero crossings in the whole sequence must be no more than one, and the envelope mean value in any time range must be zero. Then, the threshold detection is carried out for each unit. If the amplitude of the extreme value in one unit is greater than the set threshold, the unit is considered a valuable signal to be reserved. In comparison, if the amplitude is less than the threshold, the unit is considered a noise signal and set to zero. It can be expressed as:

(11) $$s^{\left(i\right)}\left(n_{j}\right)=\left\{\begin{array}{ll} s^{\left(i\right)}\left(n_{j}\right)\times \frac{\left|s^{\left(i\right)}\left(N_{j}\right)\right|-T_{i}}{\left|s^{\left(i\right)}\left(N_{j}\right)\right|}, & \left|s^{\left(i\right)}\left(N_{j}\right)\right|>T_{i}\\ 0, & \left|s^{\left(i\right)}\left(N_{j}\right)\right|\leq T_{i} \end{array}\right.,$$

where $s^{\left(i\right)}\left(n_{j}\right)$ represents the sequence which is consist of the instant values contained in the *j*th unit of the ith IMF; $s^{\left(i\right)}\left(N_{j}\right)$ is the extreme value of the sequence above; $T_{i}$ is the threshold of the ith IMF, which can be expressed as:

(12) $$T_{i}=C\sqrt{2E_{i}\mathrm{lnN}},$$

where N is the length of the IMF component and C is a constant. Assuming $E_{1}$ is the energy of the first IMF, the noise-only energy $E_{i}$ of the ith IMF can be computed directly:

(13) $$E_{i}=\frac{E_{1}}{{\beta \rho }^{i}},$$

where i = 2, 3,…, and β, ρ are parameters that mainly depend on the number of iterations. There we set C = 0.025, β = 0.719, ρ = 2.01 [17] for A = 30, which is the times of iterations;

*(6) Iterate:* Iterate A-1 times steps (3)–(5) to obtain the corresponding noise reduction versions;

*(7) Results of processing:* Add and average the noise reduction versions generated after each iteration. Figure 7 shows the signal obtained by the Iterative VMD Wavelet-Interval-Thresholding algorithm to process the phase difference signal shown in Figure 5a. Figure 7b shows the spectrum in the frequency domain of the denoised signal. Compared with the spectrum shown in Figure 5b, the spectral energy of the noise part decreases significantly. Because of the randomly altering operation of the noise component, the noises offset each other when averaging the iteration results, which is also the reason for the reduction in the noisy spectrum amplitude.

#### 2.2.5. Vital Signs Detection

The respiration rate is obtained by extracting its spectral peak value through the FFT-CZT hybrid algorithm. The algorithm mainly refers to our previous work [14], and refines the spectrum based on FFT to obtain more accurate local spectrum information. Since the amplitude of chest vibration caused by breathing is much larger than that caused by the heartbeat, the extraction of the heartbeat signal is more complex. The following are the specific steps.

*(1) Filter out the respiratory signal through a band-pass filter:* Considering the general heart-rate range, we select an elliptical band-pass filter with a passband of 0.7–3.3 Hz. The finite transmission zeros in the stopband of the elliptic filter reduce the transition region, and then an extremely steep attenuation curve can be obtained. Thus, it has a very narrow transition bandwidth;

*(2) Filter out the respiratory harmonic interference through notch filter banks:* The nonlinearity of the FMCW radar will lead to the generation of respiratory harmonics, in which the second to fourth harmonic components are within the range of humans’ heart rate, which will affect the extraction of the heart rate. Therefore, the notch filter banks designed in paper [14] are used to filter them out, and the notch frequency points are set to 2–4 times the extracted respiratory frequency above;

*(3) Heart rate judgment:* Due to the intermodulation between the breathing and heartbeat signals and the characteristic of multiple scattering points of humans’ chests, there are still frequency components other than heartbeat frequency after removing breathing and its harmonic components. There are two extraction methods:

- The FFT-CZT hybrid algorithm mentioned above
- Peek-seeking in the time domain

If $N_{0}$ is a certain sampling time at a peak in the time domain, we propose the following conditions for its amplitude $P\left(N_{0}\right)$:

(14) $$P\left(N_{0}\right)>P\left(N_{0}-1\right)>0,\;\;\;P\left(N_{0}\right)>P\left(N_{0}+1\right)>0,\;P\left(N_{0}-1\right)>P\left(N_{0}-2\right),\;\;\;P\left(N_{0}+1\right)>P\left(N_{0}+2\right),$$

where $P\left(N_{0}-1\right)$ represents the amplitude of the previous sampling point, $P\left(N_{0}+1\right)$ represents the amplitude of the next sampling point, and so on. The limitation of the above conditions can avoid the statistics of some additional spikes, which will affect the frequency estimation.

The above two methods have their advantages and limitations. Combined with the confidence of the FFT method, the threshold decision scheme of heart-rate estimation is given: if the confidence of FFT exceeds the threshold and the difference between the results of the two methods above is less than the threshold, the FFT-CZT hybrid algorithm is used for heart-rate estimation; otherwise, the peak-seeking in the time-domain is adopted. Suppose that $S_{\mathrm{FFT}}$ is the confidence of the FFT algorithm, referring to the definition of SNR, we describe this parameter as:

(15) $$S_{\mathrm{FFT}}=\frac{E_{\mathrm{peak}}}{E_{\mathrm{signal}}-E_{\mathrm{peak}}},$$

where $E_{\mathrm{signal}}$ refers to the total energy of the spectrum obtained by the extracted heartbeat signal after FFT; $E_{\mathrm{peak}}$ refers to the sum of the energy contained in the peak spectral line and its adjacent two spectral lines in the spectrum. We chose the three spectral lines mentioned above as the calculation range of the energy of the useful heartbeat signal. It is because the resolution of the FFT algorithm is limited, so the actual heart rate may not correspond to the frequency at the peak.

## 3. Experiments

**Figure 8:**
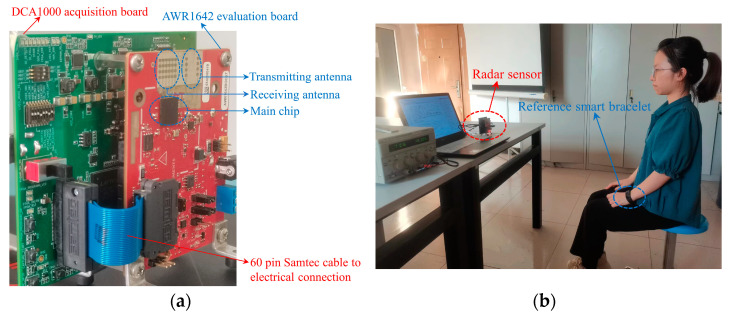
(**a**) TI AWR1642 sensor system; (**b**) Measurement scenario.

We chose the radar AWR1642 from Texas Instruments (TI) for the experiment. It is an integrated FMCW millimeter-wave sensor operating at 76–81 GHz. It integrates a high-performance DSP subsystem and ARM processor for post-processing, which can realize programming and signal processing on the chip. However, in order to further observe the experimental results and analyze the data, we hope to obtain the original data for the algorithm verification. Therefore, we used the AWR1642 evaluation board to test the target and the DCA1000 acquisition board to collect the raw radar data. Then, we imported the collected data into MATLAB for signal analysis. Figure 8a shows the sensor device we used, and the test scenario of this experiment is shown in Figure 8b. The measured person sat in front of the radar sensor while wearing a PPG+ECG smart bracelet to capture the respiratory and heartbeat rates for reference. We first verified the reliability of the smart bracelet through five groups of measurement experiments; each group lasted for 1 min. It was proved that the respiration and heartbeat rate displayed by the bracelet was no more than one bpm different from the actual value. Thus, the measured value of the smart bracelet can be taken as the reference value.

**Figure 9:**
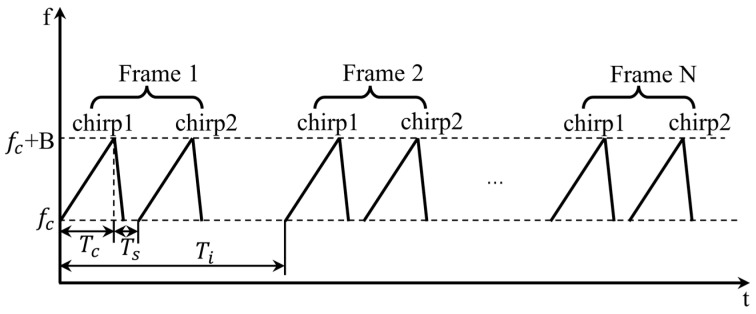
The parameter settings of the sensor.

The parameter configuration of the experiment is shown in Table 1, in which the parameters are described in Figure 9. Among them, idle time indicates the time from the end of the last chirp to the start of the next chirp. With the chirp duration and chirp slope setting, the sweeping bandwidth B of the chirp is calculated as 4 GHz. We collected 512 frames of echo data in each measurement, including two chirps in each frame, as shown in Figure 9. Only the sampled data of one chirp in each frame need to be imported into MATLAB and analyzed in the signal processing, while the other chirp is regarded as an alternative. We selected 256 frames for cardiopulmonary signal extraction. According to the frame period set, the observation duration is 12.8 s.

## 4. Results

### 4.1. Scenario Settings

A total of 11 subjects were selected for this experiment and divided into two groups. Table 2 shows the basic information of each subject, where the reference values are measured by the bracelet mentioned above. The first group had seven samples measured, in which the subjects’ respiratory and heart rates were within the generally normal range, which means that the respiratory rate is within 25 times per minute, and the heart rate is within 100 times per minute. The subjects were required to rest for at least 5 min before each measurement to avoid abnormal vital signs. During the measurements, the subjects sat on the chair in front of the sensor without intentional movement. The experimental environment was an ordinary office environment, and there were no other human targets within the sensing range of the sensor, as shown in Figure 8b. In addition, the subjects’ breathing and heart rate were measured by varying the distance to the radar sensor in the range of 0.8 m, 1 m, 1.3 m, and 1.5 m, respectively. In the second group, there were four samples. The subjects deliberately accelerated the breathing rhythm or completed 15 min of aerobic exercise to increase the respiratory and heartbeat rates, respectively, exceeding the normal range above. This group verified the applicability of the proposed algorithm.

All measurement processes lasted for 25.6 s, and then the stable data were intercepted from them with a length of 12.8 s for subsequent processing.

### 4.2. Vital Signs Waveform Recovery

**Figure 10:**
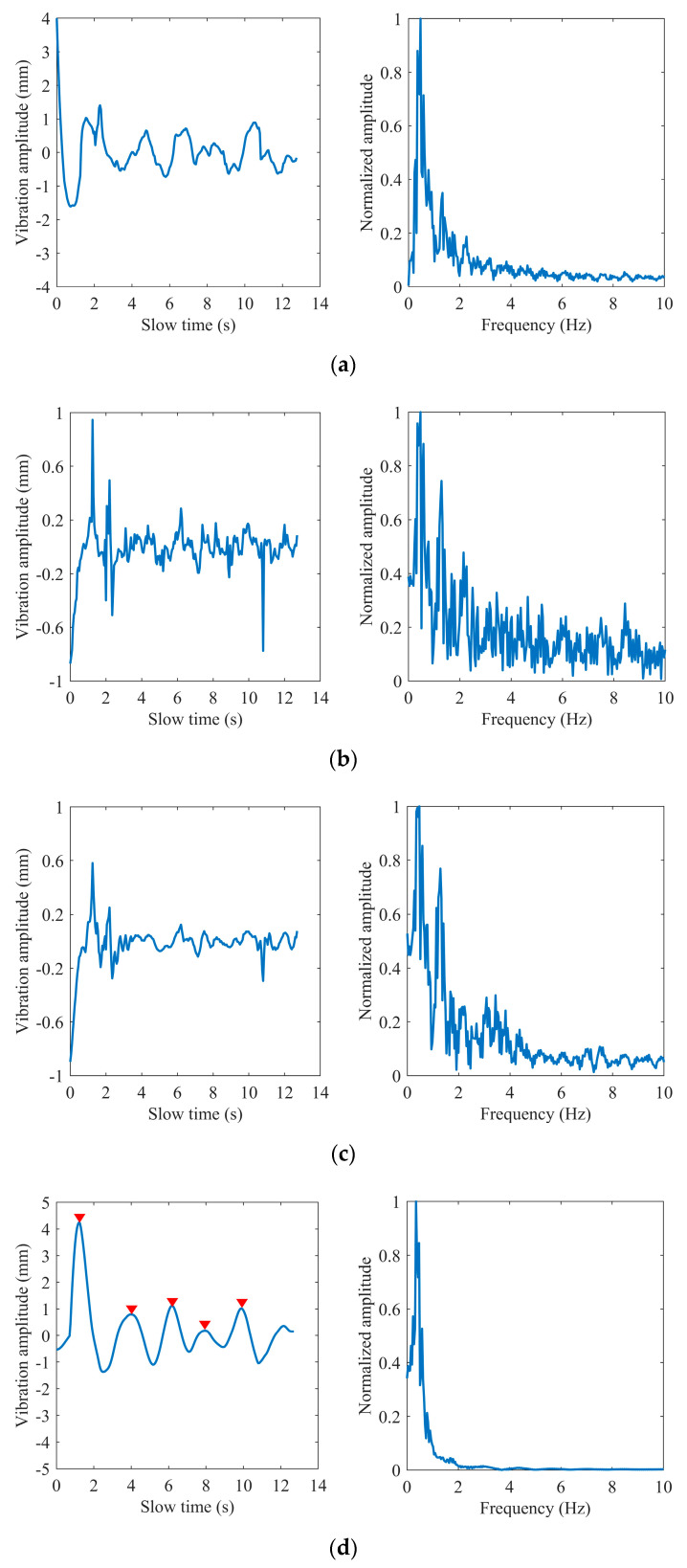

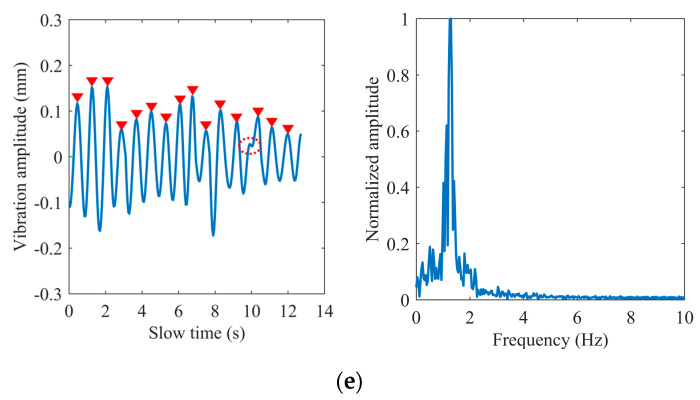
Subject #1: (**a**) extracted phase; (**b**) phase difference; (**c**) the impulse noise removal; (**d**) breathing waveform; (**e**) heart waveform.

**Figure 11:**
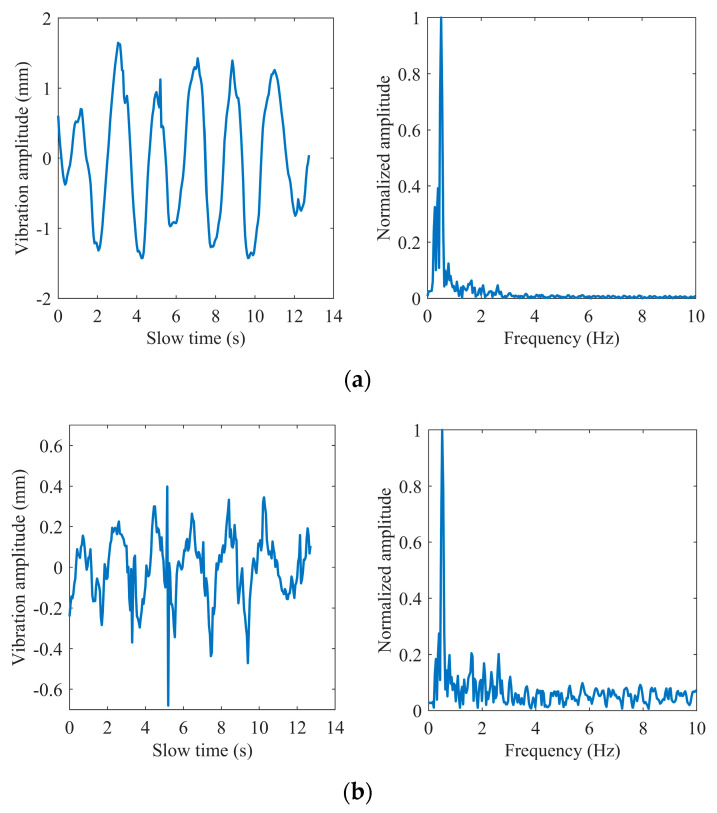

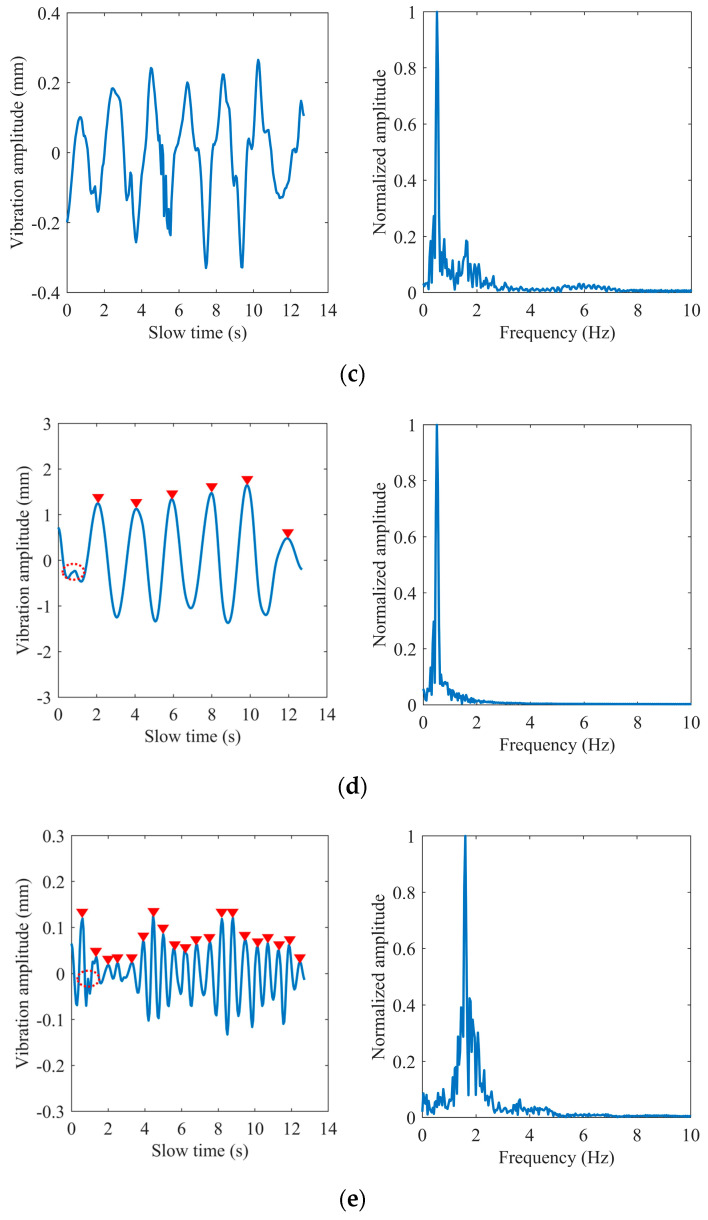
Subject #2: (**a**) extracted phase; (**b**) phase difference; (**c**) the impulse noise removal; (**d**) breathing waveform; (**e**) heart waveform.

**Figure 12:**
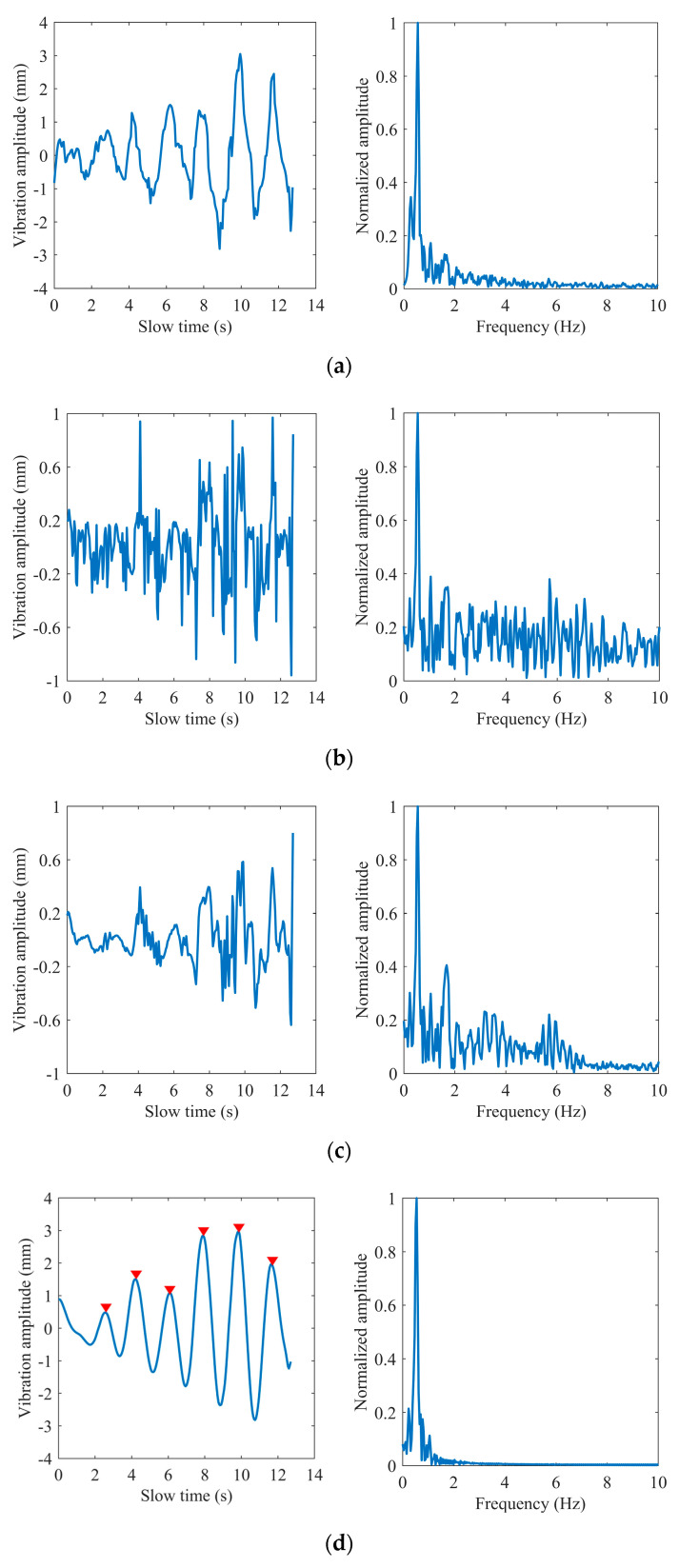

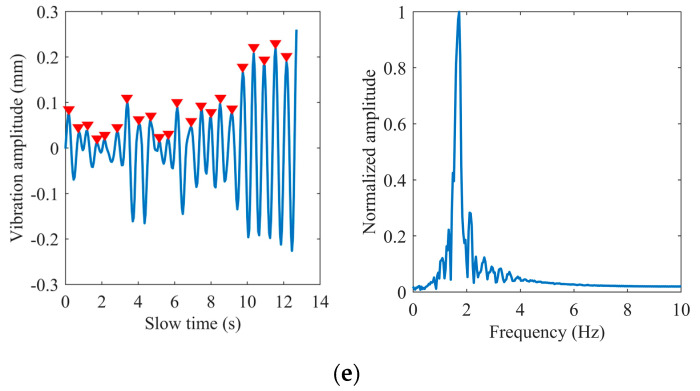
Subject #3: (**a**) extracted phase; (**b**) phase difference; (**c**) the impulse noise removal; (**d**) breathing waveform; (**e**) heart waveform.

As shown in Figure 10, Figure 11 and Figure 12, we selected three different test scenarios to show the waveforms processed in each step to verify the reliability of the proposed algorithm. The scenarios are described as follows:

Scenario #1 in the first group: the subject sat on a chair at a distance of 0.8 m in front of the radar and kept the respiration and heartbeat signs stable before the test with the respiratory and heart rate at 24 and 75 times per minute, respectively, obtained by the smart bracelet;

Scenario #2 in the second group: the subject sat still on the chair at a distance of 1.3 m in front of the radar with a deliberately increased respiratory rate at 35 times per minute;

Scenario #3 in the second group: the subject sat on the chair at a distance of 1 m in front of the radar with some routine behavioral patterns and performed aerobic exercise before the measurements to obtain a deliberately increased respiratory and heart rate at 31 and 103 times per minute.

The breathing and heartbeat signals extracted from the denoised phase difference signals by VMD decomposition operation are shown in (d) and (e) in Figure 10, Figure 11 and Figure 12. We set the number of decomposition levels to 3, and α = 2000, τ = 0, and ε = $10^{-7}$. It is not difficult to find that the waveforms of the heartbeat signal are distorted at some peaks marked by the red ellipses, which deteriorate the spectrum estimation. This is the reason for the conditions of peak judgment in peak-seeking in the time domain we proposed. Assuming that the number of peaks in the heartbeat waveform obtained in a measurement is $N_{p}$, and considering that the length of data we process each time is 256 frames, which corresponds to 12.8 s in slow time, the heartbeat rate can be expressed as:

(16) $$f_{h}=N_{p}\times \frac{1}{12.8}\;\left(\mathrm{Hz}\right).$$

### 4.3. Accuracy Analysis

Table 3 and Table 4 show the heart rate decision process in some cases of the two groups of different measurement scenarios described in Section 4.1, respectively. The relative error is calculated as follows:

(17) $$\mathrm{HR}=\left|1-\frac{f_{h}\times 60}{N_{\mathrm{ref}}}\right|\times 100\%,$$

where $f_{h}$ is the judgment result of the heart rate estimation (unit: Hz); $N_{\mathrm{ref}}$ is the reference value of the heart rate measured by smart bracelet (unit: bpm).

The effect of the Iterative VMD Wavelet-Interval-Thresholding is analyzed below. We redefined the SNR for respiratory and heartbeat signals in the frequency domain for quantitative analysis. Taking the SNR of the respiratory signal as an example, assuming that the spectral line position corresponding to the peak of its spectrum is ${\mathrm{PM}}_{0}$, its SNR can be expressed as:

(18) $$\mathrm{SNR}=10\mathrm{lg}\left(\frac{S^{2}\left({\mathrm{PM}}_{0}\right)+S^{2}\left({\mathrm{PM}}_{0}-1\right)+S^{2}\left({\mathrm{PM}}_{0}+1\right)}{S^{2}\left(f\right)-S^{2}\left({\mathrm{PM}}_{0}\right)-S^{2}\left({\mathrm{PM}}_{0}-1\right)-S^{2}\left({\mathrm{PM}}_{0}+1\right)}\right),$$

where $S^{2}\left({\mathrm{PM}}_{0}\right)$ refers to the energy contained at the peak of the signal spectrum, and $S^{2}\left(f\right)$ is the total energy of the spectrum. Table 5 shows the SNR comparison of the extracted breathing and heartbeat signals of the three subjects mentioned in Section 4.2. ${\mathrm{SNR}}_{0}$ represents the quality of the signal extracted directly without the denoising algorithm processing, and ${\mathrm{SNR}}_{1}$ represents the signal quality after denoising. The graph shows that the algorithm improves the SNR of cardiopulmonary signals of the three measured subjects. The SNR of the respiratory signal increases by 1.8922 dB on average, and that of the heartbeat signal increases by 1.441 dB on average, which are better than the SNR improvement in paper [20], with an average increase of 1.11 dB and 0.99 dB, respectively.

**Figure 13:**
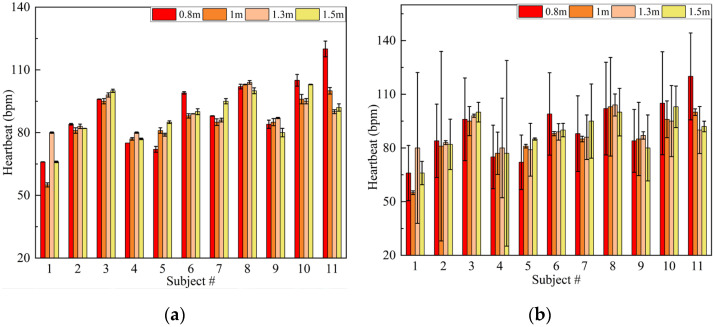
Experimental results of the heartbeat (**a**) with and (**b**) without Iterative VMD Wavelet-Interval-Thresholding algorithm. (The bars represent the reference values and the error bars represent the absolute errors of the measurement values obtained by the two algorithms compared to the reference values).

Because the amplitude of the chest cavity vibration caused by respiration is relatively large, it is not easy to be disturbed by noise. Thus, we only analyzed the detection accuracy of the heartbeat rate here. Figure 13 shows the comparison of heartbeat rate accuracy with and without the Iterative VMD Wavelet-Interval-Thresholding algorithm. It can be found that the proposed noise reduction algorithm has good adaptability to different test objects and distances. The accuracy is obviously better than without the use of this algorithm. Considering the operation of randomly altering noise sequences in the iterative algorithm, we repeatedly processed each group of collected data 20 times and calculated the solution time each time. The average computation time of the above two algorithms is 5.78 s and 2.36 s, respectively. This time difference is acceptable, which makes it possible for high-precision real-time detection.

**Figure 14:**
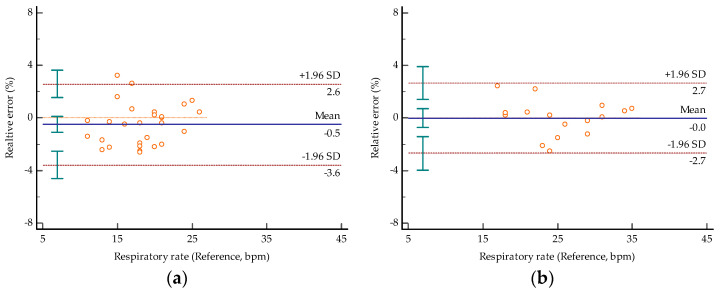

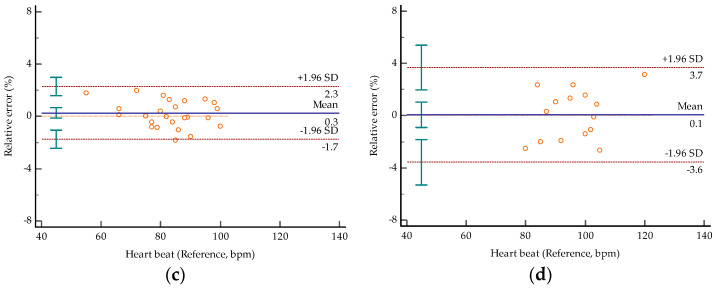
Bland–Altman plots of the values obtained by the proposed method against the reference values obtained by the smart bracelet: (**a**) respiratory rate of Group #1; (**b**) respiratory rate of Group #2; (**c**) heart rate of Group #1; (**d**) heart rate of Group #2.

Figure 14 shows the Bland–Altman plots of respiratory and heart rates obtained by the proposed method against their reference values, respectively. The X axis is the reference values, and the Y axis is the relative error of the measured values relative to the reference values. The figure shows the limits of agreement at −3.6% and 2.6% for respiratory rates and at −1.7% and 2.3% for heart rates in the first group, while in the second group the limits of agreement are at −2.7% and 2.7% for respiratory rates and at −3.6% and 3.7% for heart rates. Comparing the measured value of respiratory rates with the reference value, the average relative errors of the two groups are 1.33% and 1.01%, respectively. The average relative errors of the heart rates are 0.86% and 1.96%, respectively. The average values mentioned above are different from the mean values marked in Figure 14, which are based on the absolute value and thus more referential in the degree of characterization accuracy. It can be found that the heart rate detection accuracy of the second group is decreased but can be ignored. This means that our proposed algorithm is capable of abnormal situation monitoring.

Table 6 lists the comparison results with other works. It can be found that under basically the same test scenario, only one FMCW radar sensor can detect respiration and heart rate with a higher accuracy.

## 5. Discussion

**Figure 15:**
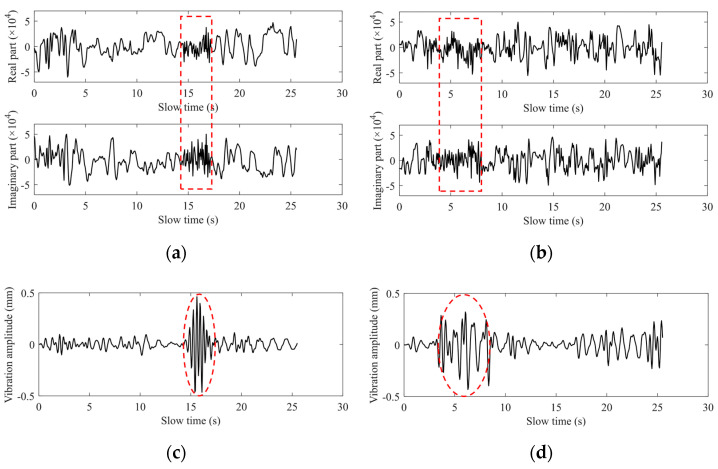
(**a**) The complex data at the selected range bin in a coughing situation; (**b**) The complex data at the selected range bin in the case of arm swinging; (**c**) The heartbeat waveform of coughing scene; (**d**) The heartbeat waveform of arm-swing scene.

This paper introduces a high-precision vital-signs detection algorithm based on millimeter-wave radar sensors. In practical situations, however, the monitored person does not stay completely still. So, next we discuss the applicability of the proposed algorithm in the non-stationary cases. It is mainly divided into two situations: small-scale micro-movements and large-scale and fast random body movement (RBM). The former includes coughing, sneezing, and arm swinging, while the latter refers to behaviors such as walking around. The difference between the two is whether there is a significant change in the displacement of the chest wall over time. Figure 15 shows the signal fluctuation detected when the subject’s body moves slightly. The marked parts in Figure 15a,c show the influence of cough behavior on the waveform, while the marked parts in (b) and (d) show the influence of arm-swing behavior on the waveform. It can be found that the selected distance window can still extract the phase signal containing the vital signs’ information because the body’s micro-motion behavior does not bring about significant changes in the position of the chest wall. The heartbeat signal extracted by this algorithm only has waveform distortion in the short-term window corresponding to the fretting behavior, and the other part in the entire detected time window is not affected. Therefore, the distorted part of the heartbeat waveform can be detected by the threshold and eliminated, and then spectrum estimation can be performed to obtain the heart rate. Since RBM can lead to changes in the chest wall position, the range-window selection algorithm adopted in this paper is no longer applicable. To eliminate its effect on chest wall vibrations caused by cardiorespiratory activity, two Doppler radar systems [22] or a radar system with a camera [23] were used to remove RBM. In addition, an adaptive noise cancellation technique combined with the polynomial fitting was proposed to offset the influence of RBM on the extraction of small vital sign signals [10]. We expect to implement more efficient RBM removal algorithms to reduce costs in future work.

Meanwhile, deep learning-based technology has also been studied and applied to the detection of vital signs’ information in order to adapt to complex and changeable scenarios. At present, the more mature application is mainly to distinguish whether the target’s respiratory or heart rate is abnormal with high precision. Yin. W et al. proposed using convolutional neural networks independent of each other to learn the signals collected by ECG and the UWB radar and then performed the comprehensive analysis in a cascaded way to achieve mutual complement of biological and physical characteristics of the heartbeat [24]. Li. J proposed to convert the constructed heartbeat waveform into an image and used VGG for identification [25]. For heart rate monitoring, convolutional neural network recognition technology based on the camera-based remote photoplethysmography method [26] and the fiber-optic sensor [27] has achieved high-precision detection. However, there are few related studies based on radar sensors, which may be because deep learning currently mainly relies on image training, and it is difficult to convert the electrical signals propagated by radar into images, which is also a challenge for the future.

## 6. Conclusions

In this paper, we discussed a systematic method for respiration and heartbeat monitoring based on a FMCW millimeter wave radar. Two scenarios with 11 human subjects at different distances were performed and studied. With the 25.6 s short-time monitoring in each experiment, a window of 12.8 s was randomly selected for signal processing and vital signs’ information extraction. The results show that the average accuracy of respiratory and heart rate estimation is not less than 98% using the FMCW sensor and the proposed processing algorithm in the test scenario where subjects are stationary. The possible noise contained in the test process and the corresponding noise removal measures were discussed. The experiments showed that the SNR of the vital signals can be increased by using the Iterative VMD Wavelet-Interval-Thresholding algorithm. In addition, the decision method based on spectrum refinement and peek-seeking in the time domain also helps to improve the accuracy of rate estimation. We envision that this approach could be applied to various practical situations, such as home health-monitoring for the elderly, medical assistance for burn victims, and driver monitoring systems.

## Figures and Tables

**Table 1:** FMCW signal parameters for vital signs measurements.

Parameter	Value
Chirp starting frequency, $f_{c}$	77 GHz
Chirp duration, $T_{c}$	50 μs
Chirp slope, K	80 MHz/μs
Idle time, $T_{i}$	7 μs
ADC sampling rate, $F_{s}$	6.4 Msps
Frame period, $T_{s}$	50 ms
Sample points number in each chirp, $N_{\mathrm{samples}}$	256
Transmitting antenna	${\mathrm{Tx}}_{0}$
Receiving antenna	${\mathrm{Rx}}_{0}$
Output power	12.5 dBm

**Table 2:** Subjects’ basic data.

Group #	Subject #	Basic Health Information			Distance (m)	Reference Value (bpm)	
		Sex	Age	SD		Breathing	Heartbeat
1	1	Female	21	Normal breathing and heart rate	0.8	11	66
					1	21	55
					1.3	18	80
					1.5	26	66
	2	Female	24	Normal breathing and heart rate	0.8	20	84
					1	15	81
					1.3	25	83
					1.5	18	82
	3	Male	31	Normal breathing and heart rate	0.8	15	96
					1	17	95
					1.3	14	98
					1.5	24	100
	4	Female	24	Normal breathing and heart rate	0.8	16	75
					1	13	77
					1.3	18	80
					1.5	18	77
	5	Female	26	Normal breathing and heart rate	0.8	11	72
					1	20	81
					1.3	21	79
					1.5	18	84
	6	Female	22	Normal breathing and heart rate	0.8	13	99
					1	24	88
					1.3	24	89
					1.5	21	90
	7	Male	24	Normal breathing and heart rate	0.8	21	88
					1	19	85
					1.3	20	86
					1.5	17	95
2	1	Female	23	Rapid heartbeat with deliberately rapid breathing in some cases	0.8	22	102
					1	31	103
					1.3	18	104
					1.5	18	100
	2	Male	24	Normal heartbeat with deliberately rapid breathing in some cases	0.8	26	84
					1	23	85
					1.3	34	87
					1.5	31	82
	3	Male	24	Rapid heartbeat and deliberately rapid breathing in some cases	0.8	21	108
					1	29	94
					1.3	35	95
					1.5	29	103
	4	Male	29	Normal breathing with deliberately rapid heartbeat in some cases	0.8	15	120
					1	17	100
					1.3	25	90
					1.5	24	92

**Table 3:** Heart rate decision result of group #1.

Subject #	Range (m)	Reference (bpm)	Measurement (Hz)		Judgment (Hz)	Relative Error
			FFT-CZT	Peek-Seeking		
1	0.8	66	1.0989	1.0547	1.0989	0.1%
	1.3	80	1.8732	1.3281	1.3281	1.27%
2	0.8	84	3.5245	1.4063	1.4063	0.45%
	1.3	83	1.3657	1.4063	1.3657	0.37%
3	1.3	98	1.6165	1.6016	1.6165	1.03%
	1.5	100	1.9058	1.6797	1.6797	0.78%
4	1	77	1.2939	1.3672	1.2939	0.82%
	1.3	80	2.4911	1.3281	1.3281	0.39%
5	0.8	72	1.1765	1.25	1.1765	1.96%
	1.5	84	2.7039	1.4063	1.4063	0.45%
6	0.8	99	1.9977	1.6404	1.6404	0.58%
	1	88	1.4493	1.4453	1.4493	1.18%
7	0.8	88	1.4682	1.4844	1.4682	0.11%
	1.5	95	1.2390	1.5625	1.5625	1.32%

**Table 4:** Heart rate decision result of group #2.

Subject #	Range (m)	Reference (bpm)	Measurement (Hz)		Judgment (Hz)	Relative Error
			FFT-CZT	Peek-Seeking		
1	0.8	102	2.7029	1.7188	1.7188	1.11%
	1.5	100	1.6901	1.4844	1.6901	1.41%
2	1	87	1.1084	1.4453	1.4453	0.32%
	1.5	82	1.7166	1.3672	1.3672	0.04%
3	0.8	108	0.9375	1.7969	1.7969	0.17%
	1	94	2.3318	1.5625	1.5625	0.27%
4	0.8	120	1.9373	1.4844	1.9373	3.14%
	1.3	90	2.4292	1.4844	1.4844	1.04%

**Table 5:** SNR comparison before and after Iterative VMD Wavelet-Interval-Thresholding.

Subject #	Breathing Signal		Heartbeat Signal	
	$SBR_{0}\;(\mathrm{dB})$	$SBR_{1}\;(\mathrm{dB})$	$SBR_{0}\;(\mathrm{dB})$	$SBR_{1}\;(\mathrm{dB})$
1	−3.1784	−2.7656	−0.8765	0.3583
2	7.8689	8.8274	−2.8444	−1.0186
3	0.1020	4.4074	−3.0217	−1.7594

**Table 6:** Comparison of the proposed algorithm with other works.

Ref. No.	Output Power (mW)	Error of Breathing Rate	Error of Heartbeat Rate
[12]	17.8	Not mentioned	3.02%
[15]	15.8	6%	20%
[21]	0.5	6.89%	8.09%
This work	17.8	1.33%	1.96%

## Data Availability

Data available on request due to privacy restrictions.
